# Loss of Multi-Epitope Specificity in Memory CD4^+^ T Cell Responses to *B. Pertussis* with Age

**DOI:** 10.1371/journal.pone.0083583

**Published:** 2013-12-31

**Authors:** Wanda G. H. Han, Inonge van Twillert, Martien C. M. Poelen, Kina Helm, Jan van de Kassteele, Theo J. M. Verheij, Florens G. A. Versteegh, Claire J. P. Boog, Cécile A. C. M. van Els

**Affiliations:** 1 Centre for Immunology of Infectious Diseases and Vaccines, National Institute for Public Health and the Environment, Bilthoven, The Netherlands; 2 Department of Statistics, Mathematical Modelling and Data Logistics, National Institute for Public Health and the Environment, Bilthoven, The Netherlands; 3 Julius Center Health Sciences and Primary Care, University Medical Center Utrecht, Utrecht, The Netherlands; 4 Department of Pediatrics, Groene Hart Ziekenhuis, Gouda, The Netherlands; 5 Institute for Translational Vaccinology, Bilthoven, The Netherlands; Leiden University Medical Center, The Netherlands

## Abstract

Pertussis is still occurring in highly vaccinated populations, affecting individuals of all ages. Long-lived Th1 CD4^+^ T cells are essential for protective immunity against pertussis. For better understanding of the limited immunological memory to *Bordetella pertussis*, we used a panel of Pertactin and Pertussis toxin specific peptides to interrogate CD4^+^ T cell responses at the epitope level in a unique cohort of symptomatic pertussis patients of different ages, at various time intervals after infection. Our study showed that pertussis epitope-specific T cell responses contained Th1 and Th2 components irrespective of the epitope studied, time after infection, or age. In contrast, the breadth of the pertussis-directed CD4^+^ T cell response seemed dependent on age and closeness to infection. Multi-epitope specificity long-term after infection was lost in older age groups. Detailed knowledge on pertussis specific immune mechanisms and their insufficiencies is important for understanding resurgence of pertussis in highly vaccinated populations.

## Introduction

Whooping cough or pertussis is an acute infection of the upper respiratory tract that is most severe in young children [1]. The disease is caused by the *Bordetella (B.) pertussis* bacterium and infants worldwide are vaccinated against pertussis since the 1950's. Despite high vaccination coverage, resurgence of pertussis was observed in many countries since the 1990's, affecting not only non- or partially vaccinated neonates but also adolescent, adult and elderly vaccinees [2]–[7]. Estimates of the duration of immunity against *B. pertussis* range from 4–12 years after vaccination and 4–20 years after infection, indicating insufficient long-term effectiveness of pertussis-specific adaptive responses [8]. The emergence of new variants of *B. pertussis* may enhance waning effectiveness of pertussis immunity, due to increasing mismatch between vaccine- and circulating strains, in which polymorphisms in coding or promotor regions of important virulence factors and even functional deletion of vaccine antigens are found to occur [9]–[11]. Therefore, a relative narrow response to only a few pertussis antigens present in acellular pertussis vaccines (aP), could also play a role in the current sub-optimal long-term immunity against pertussis and increased incidence of whooping cough [8], [12].

In addition to antibodies, pertussis-specific Th1 and Th17 type CD4^+^ T cells are essential for protective immunity against *B. pertussis* challenge in mice [13]–[21]. Previous human studies indicate induction of Th1 and Th2 type pertussis-specific T cell responses after aP vaccination, while Th1 or Th17 type responses are seen after infection with *B. pertussis* [22]–[25]. Besides the cytokine differentiation of the CD4^+^ T cell response, also the magnitude and finespecificity determines it's effectiveness. A broad response in which pathogen-specific CD4^+^ T cells are responding to multiple epitopes and multiple antigens, is usually regarded important for protective immunity [26]. Furthermore, optimal T cell memory potential is considered to involve good self-renewal capacity under steady state conditions and potent lymphoproliferative capacity in a recall response [27], [28].

Knowledge on the breadth and quality of pertussis-specific CD4+ T cell responses at the epitope level is important yet lacking. This is not in the least because healthy blood donors contain these cells at near or below detection limit. Most studies investigating pertussis-specific CD4^+^ T cell responses use PBMC from vaccinees and whole P.69 Pertactin (P.69 Prn) or Pertussis toxin (Ptx) vaccine antigens, which strictly speaking are not indicative for the breadth and quality of CD4^+^ T cell response to single epitopes [22]–[25], [29]–[31]. In this study we interrogate quantitative and qualitative aspects of pertussis epitope specific CD4^+^ T cell responses in *B. pertussis* exposed individuals, to identify eventual biomarkers of waning immunity. Recently, we identified a panel of P.69 Prn and Ptx Subunit S1 (PtxS1) CD4^+^ T cell epitopes. In a unique clinical cohort of symptomatic pertussis patients, sampled at various time intervals after their laboratory confirmed diagnosis, and household contacts, we assessed the lymphoproliferative capacity, cytokine profile and epitope breadth of Prn- and Ptx-specific CD4^+^ T cell responses and these features were analyzed in relation to age and time since infection.

## Results

### Specific T cell proliferation to Prn- and Ptx-peptides

**Figure 1:**
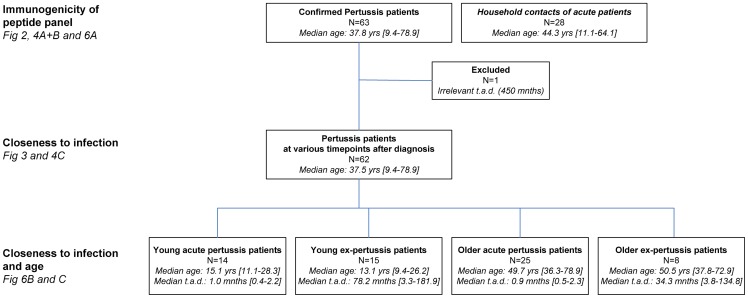
Flowchart of study populations interrogated, as part of an observational study. Symptomatic pertussis patients, and household contacts, were recruited based on the information of their laboratory confirmed diagnosis of *B. pertussis* infection provided by General Practitioners and Pediatricians. Subsets of participants were stratified based on study population, age and time after diagnosis (t.a.d.) to analyse their responsiveness for the different research questions. Median age in years (yrs) and median t.a.d. in months (mnths) are indicated.

**Figure 2:**
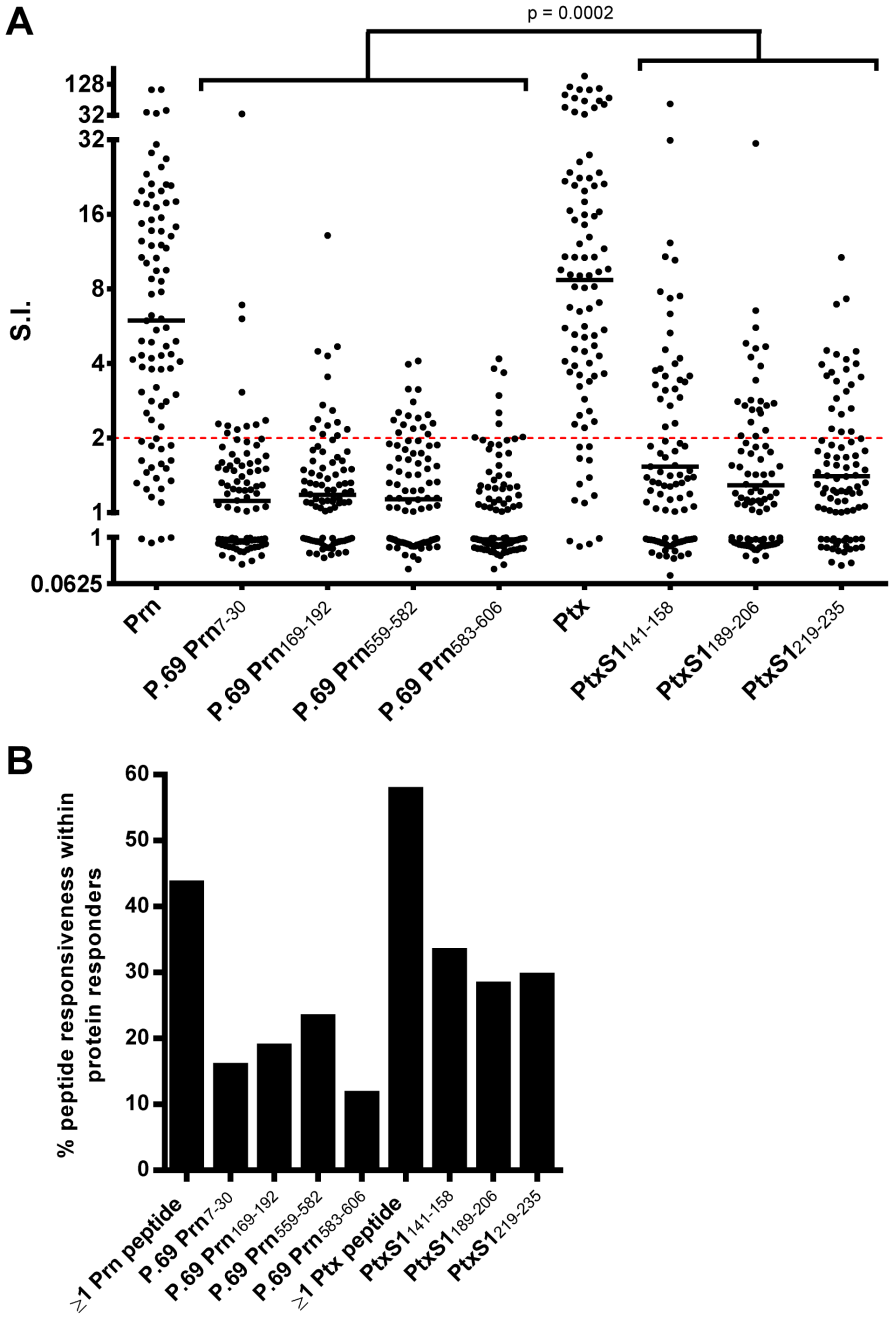
Immunogenicity of the Prn- and Ptx-peptide panel. Fresh PBMC of (ex-)pertussis patients and household contacts (n = 91) were stimulated with 1 µM peptide or 1 µg/ml protein for 7 days and [^3^H]thymidine incorporation was assed in the last 18 hours. The responsiveness of participants to PHA was 100% (Data not shown). (A) Epitope specific responsiveness shown as Stimulation Index (S.I. = geomean CPM peptide/geomean CPM medium) of (ex-)pertussis patients and their household contacts. Epitope specific responses with a S.I.≥2 were regarded as positive. Lines indicate geometric means. (B) Responsiveness to the tested Prn- or Ptx- peptides (S.I.≥2) in participants with proliferative responses to Prn (n = 69) or Ptx (n = 78) protein.

Synthetic peptides representing four Prn and three Ptx CD4^+^ T cell-epitopes were selected in our peptide panel based on identification studies by our group and others (Table 1). The selected sequences are proven CD4^+^ T cell epitopes, since these were identified either by conventional T cell cloning procedures in our group or by others [32], [33] and MHC class II blocking of those CD4^+^ T cell clones (Figure S1), or by unconventional peptide elution methods using affinity purified MHC class II molecules, operational in our group (Table 1). To analyze the immunogenicity of our peptide panel irrespective of clinical parameters, lymphoproliferative responses to these peptides were assed in PBMC from all participants in our clinical study (n = 91) including (ex-)patients and household contacts, since all these donors may be primed for these epitopes (due to infection or sub-clinical infection or vaccination) (Figure 1). The responder frequencies to whole Prn or Ptx protein stimulation were 75.8% and 85.7% (Figure 2A), respectively, indicating that in these participants Prn- and Ptx-epitope specific responses will be present. Based on binding motif analysis, all Ptx specific epitopes PtxS1_141–158_, PtxS1_189–206_, and PtxS1_219–235_, and P.69 Prn_583–606_ had relatively high prediction scores for binding to multiple HLA–DR alleles, as compared to P.69 Prn_7–30_, P.69 Prn_169–192_ and P.69 Prn_559–582_ (Figure S2). Lymphoproliferative analysis of PBMC nevertheless showed immunogenicity of all seven Prn- and Ptx-epitopes (Figure 2A). Since the S.I. of the [^3^H]thymidine assay has been found to correlate well with flowcytometric CFSE dilution and Blast analysis of proliferating T cell cultures, S.I. values reflect the magnitude of the T cell response [34], [35]. Responsiveness to single Prn or Ptx epitopes was found in 11.6% (P.69 Prn_583–606_) to 33.3% (PtxS1_141–158_) of the participants responding to whole Prn or Ptx protein, respectively (Figure 2B). The responder frequencies to ≥1 Prn-epitopes and ≥1 Ptx-epitopes were 43.5% and 57.7% of the participants responding to Prn or Ptx protein, respectively (Figure 2B), indicating that our peptide panel does not cover the whole Prn and Ptx response. Overall, lymphoproliferative responsiveness to Ptx-epitopes is higher than to Prn-epitopes in our panel (Figure 2A). Although the synthetic peptide panel does not cover the whole Prn and Ptx response, our peptide panel is able to identify half of the Prn- and Ptx-responding donors (Figure 2B) and is therefore a useful tool to characterize Prn- and Ptx-specific CD4^+^ T cell responses at the single epitope specificity level.

**Table 1:** Prn- and Ptx-peptide panel.

Epitope	Sequence	Identification Method	References
P.69 Prn_7–30_	IVKTGERQHGIHIQGSDPGGVRTA	CD4^+^ T cell clone	[51]
P.69 Prn_169–192_	LRDTNVTAVPASGAPAAVSVLGAS	MHC class II elution	van Els, unpubl. data
P.69 Prn_559–582_	PEAPAPQPPAGRELSAAANAAVNT	CD4^+^ T cell clone	van Els, unpubl. data
P.69 Prn_583–606_	GGVGLASTLWYAESNALSKRLGEL	MHC class II elution	van Els, unpubl. data
PtxS1_141–158_	IRRVTRVYHNGITGETTT	CD4^+^ T cell clone	van Els, unpubl. data [32], [33]
PtxS1_189–206_	GTLVRIAPVIGACMARQA	CD4^+^ T cell clone	[33]
PtxS1_219–235_	AGEAMVLVYYESIAYSF	CD4^+^ T cell clone	[32]

### Influence of closeness to infection on the lymphoproliferative responsiveness to Prn- and Ptx-peptides

**Figure 3:**
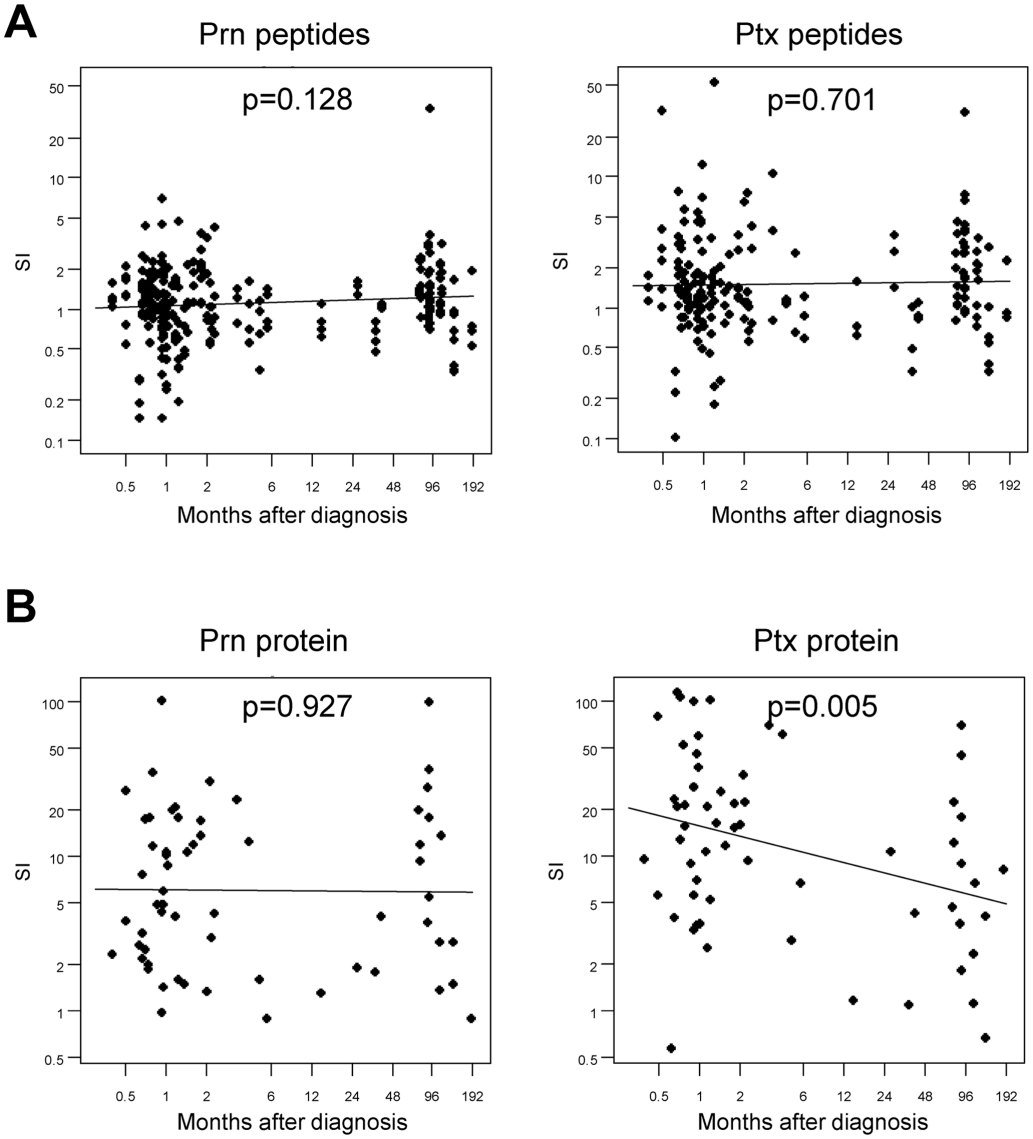
Specific lymphoproliferative responsiveness to single Prn and Ptx peptides and whole protein. Fresh PBMC were stimulated with 1 µM peptide or 1 µg/ml protein for 7 days and [^3^H]thymidine incorporation was assessed in the last 18 hours. (A) Epitope specific responsiveness shown as stimulation index (S.I. = geomean CPM peptide/geomean CPM medium) of the proliferative responses from (ex-)pertussis patients (n = 62) in correlation with months after diagnosis is plotted. The left panel shows the S.I. of P.69 Prn_7–30_, P.69 Prn_169–192_, P.69 Prn_559–582_ and P.69 Prn_583–606_ epitope responses. The right panel shows the S.I. of PtxS1_141–158_, PtxS1_189–206_, and PtxS1_219–235_ epitope responses. (B) Protein specific responsiveness to Prn (left panel) and Ptx (right panel) shown as S.I. of the proliferative responses from (ex-)pertussis patients (n = 62) in correlation with months after diagnosis is plotted. The regression lines and p-values are indicated and the slopes were tested to be equal to zero using a t-test.

An important effective feature of pathogen specific CD4^+^ T cell responses is their maintenance in time. To analyze this cross-sectionally, epitope-specific lymphoproliferative responsiveness was studied in pertussis patients (Figure 1) as a function of time after diagnosis (Figure 3A). When given as S.I., lymphoproliferative responsiveness to Prn-epitopes and whole Prn antigen measured from as early as 0–1.5 months until years after diagnosis did not significantly alter, suggesting that the Prn-specific CD4^+^ T cell responses in our overall panel had a typical maintenance level which seems stable for years (Figure 3A and B, left panels). Also Ptx-epitope specific CD4^+^ T cell responses seemed to be stable over time (Figure 3A, right panel). Yet lymphoproliferative responses to whole Ptx-antigen were high shortly after infection and did significantly decrease with time elapsed after infection (Figure 3B, right panel). Altogether, these results indicate that significant pertussis-specific CD4^+^ T responses can still be detected years after pertussis infection. In general, the magnitude of the proliferative responsiveness to the whole Ptx protein diminished with time after infection while the magnitude of whole Prn and Ptx- and Prn-epitope proliferative responses remained stable.

### Cytokine profile of Prn- and Ptx-epitope specific T cell responses after pertussis infection

**Figure 4:**
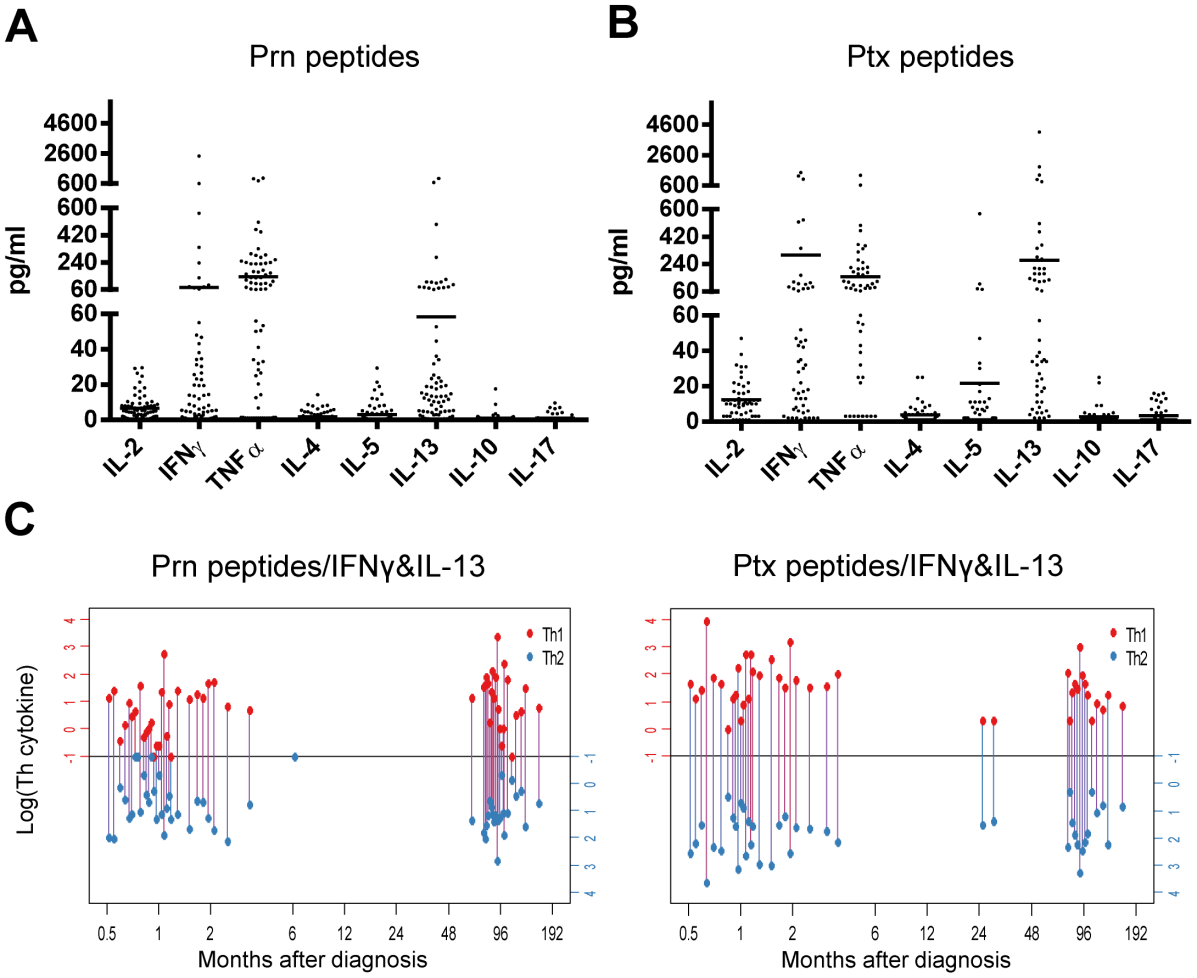
Prn- and Ptx-epitope specific cytokine profiles in relation to time after infection. Supernatants were collected from PBMC cultures of (ex-)pertussis patients with S.I.≥2 to the Prn- and Ptx-peptide panel and cytokine concentrations were analyzed by luminex. (A and B) Cytokine concentrations in supernatants from PBMC cultures of participants with positive proliferative responses (S.I.≥2) after stimulation with various Prn- (A) or Ptx-peptides (B). (C) Red and blue bullets connect levels of Th1 (IFNγ, red axis) and Th2 (IL-13, blue axis) cytokines (log10 transformed) found in single supernatants of epitope responses of individual (ex-)pertussis patients in relation to time after infection, overlapping lines were nudged for visualization. No significant trends between were observed between log(Th) and log(closeness to infection) (p-values: Prn peptides - IFNy: 0.064, Prn peptides - IL13: 0.090, Ptx peptides - IFNy: 0.126, Ptx peptides - IL13: 0.154).

To assess the quality of the pertussis-specific CD4^+^ T cell responses and Th subsets involved, we analyzed in a multiplex assay the concentration of various T cell cytokines in the supernatants from day 6 PBMC cultures from all participants in our clinical study (Figure 1) with S.I. ≥2 to a peptide from our Prn- or Ptx- peptide panel (Figure 4A). Predominantly, IFNγ and TNFα (typical Th1 cytokines) and IL-13 and some IL-5 (typical Th2 cytokines) were detected in the supernatants of the cumulative Prn-peptide responsive wells (Figure 4A). No significant epitope-specific IL-17 and IL-10 production was measured in responsive wells. Similar observations were made for the cumulative Ptx-peptide responsive wells (Figure 4B). Since we detected both Th1 and Th2 cytokines in the Prn- and Ptx-epitope specific responses, we were interested whether some epitope responses of individual patients were producing Th1 cytokines while others produced Th2 cytokines. We plotted the epitope-specific IFNγ/IL-13 responsiveness of individual pertussis patients (with an epitope proliferative response of S.I. ≥2) against time elapsed since diagnosis (Figure 4C) grouped as Prn peptide responses (Figure 4C, left panel) or grouped as Ptx peptide responses (Figure 4C, right panel). These data indicate that a mixed Th1/Th2 response to a single epitope can be detected in a single donor. Other dual Th1/Th2 cytokine combinations, i.e. IFNγ/IL-5, TNFα/IL-5 and TNFα/IL-13, showed similar trends for both Prn- and Ptx peptide panels (data not shown). It is clear that although a small number of individuals' epitope responses were Th1 only or Th2 only, the majority of the Prn- and Ptx-epitope responses in (ex-)pertussis patients have both a Th1 and Th2 component (Figure 4C). Such dual Th1/Th2 cytokine responsiveness was not dependent on time after infection (Figure 4C).

**Figure 5:**
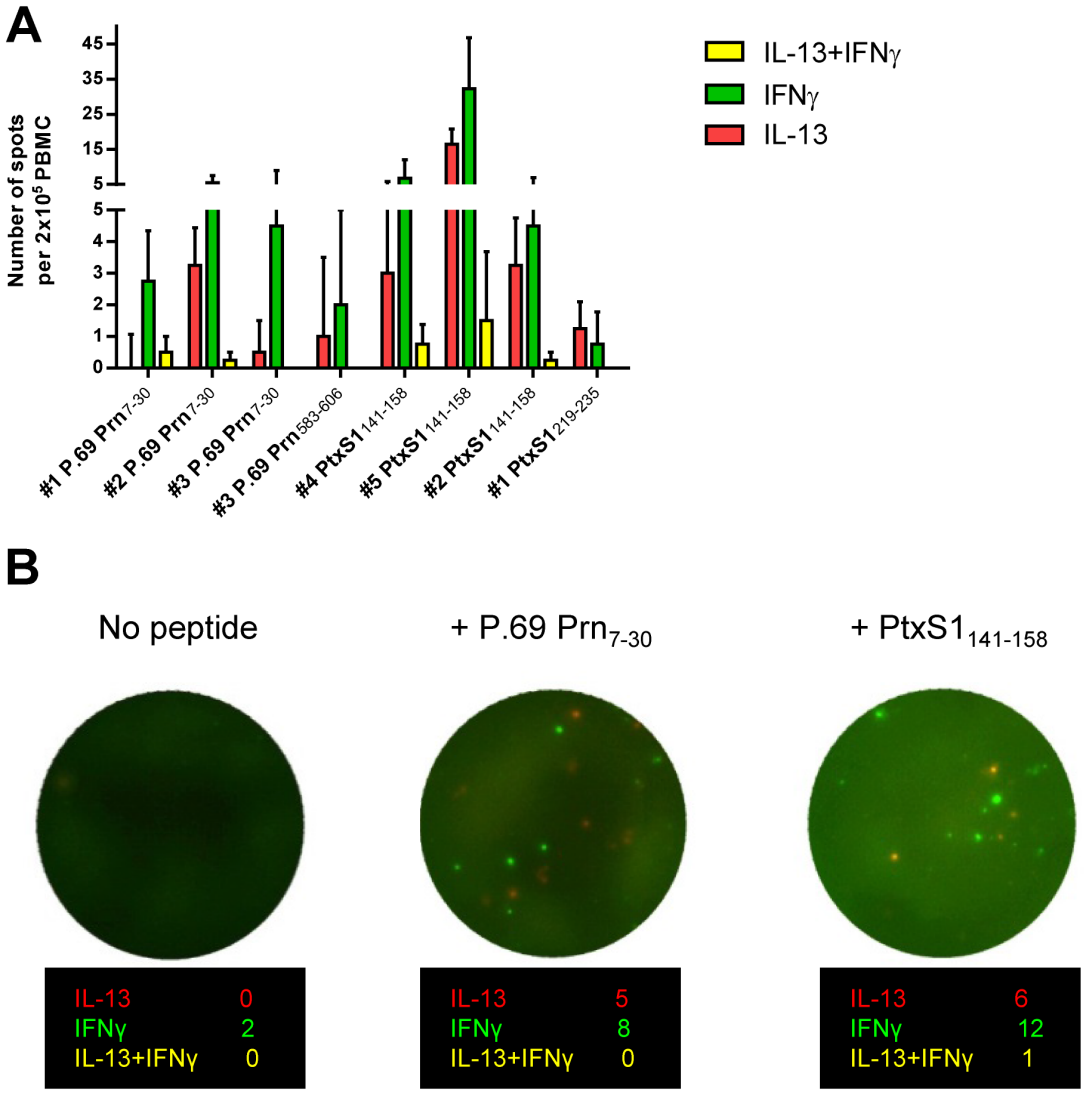
Prn- and Ptx-epitope specific Th1/Th2 mixed cytokine responses. PBMC of peptide-responding participants (n = 5, indicated as #1–#5) were stimulated with 1 µM individual Prn- or Ptx-peptides or medium, in the presence of anti-CD28 monoclonal (1 µg/ml) for 48 hours at 37°C. IFNγ^+^, IL-13^+^ and IFNγ^+^IL-13^+^ cells were identified with Fluorospot. (A) The average number of Prn and Ptx peptide-specific IFNγ^+^, IL-13^+^ and IFNγ^+^IL-13^+^ cells per 2×10^5^ PBMC in five tested participants are indicated. (B) Example of Fluorospot wells obtained from a P.69 Prn_7–30_ and PtxS1_141–158_ responsive participant.

Hence, consistent mixed Th1/Th2 cytokine production was detected after PBMC stimulation with individual CD4^+^ T cell peptides, suggesting either that multiple clonal CD4^+^ T cell populations for a single specificity with either a Th1 or a Th2 cytokine profile coexist, or that single pertussis-epitope specific CD4^+^ T cells are capable of producing Th1 and Th2 cytokines simultaneously. This was further investigated at the single cell level by Fluorospot, using frozen cells [36]. Collective data from five tested participants known to respond to peptide stimulation indicate that CD4^+^ T cell populations specific for one single epitope are mixed in cytokine profile, with the majority of epitope-specific CD4^+^ T cells producing at one given moment either Th1 or Th2 cytokines (Figure 5A and B). In case IFNγ^+^IL-13^+^ double positive spots were detected, it comprised only a small (<5%) percentage of the antigen-specific IFNγ- and IL-13-producing cells of the five tested participants (Figure 5A). Together, supernatant analysis of pertussis epitope-specific PBMC stimulations indicate the presence of stable mixed Th1/Th2 cytokine profiles in time after infection. Thus, these pertussis epitope-specific CD4^+^ T cell responses are not fixed to one Th cytokine profile.

### Multi-epitope specificity in pertussis-specific CD4^+^ T responses

**Figure 6:**
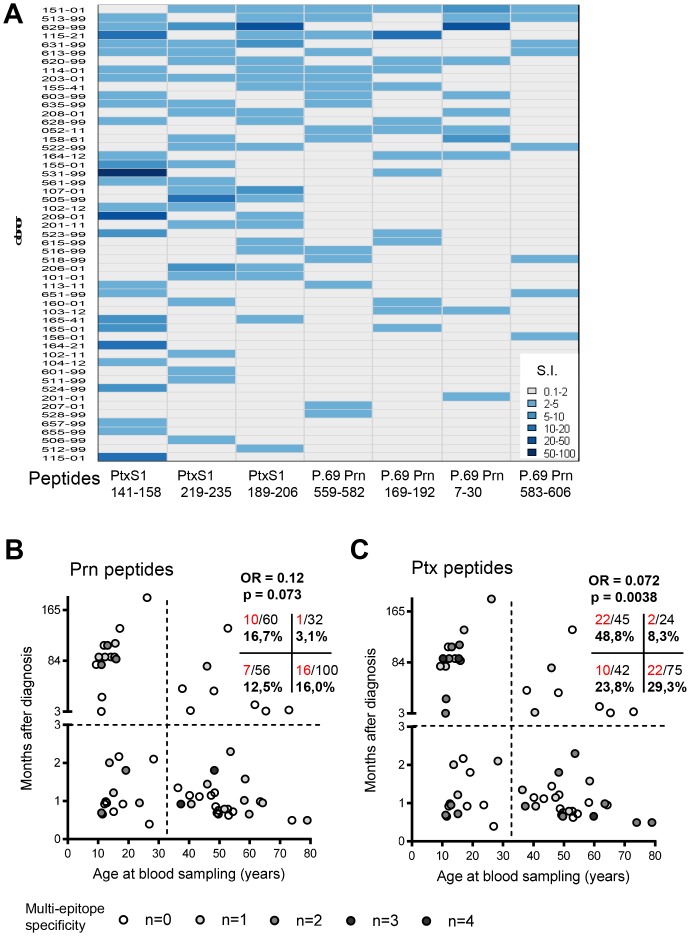
Prn- and Ptx-epitope multispecificity in individual participants. Fresh PBMC were stimulated with 1 µM peptide for 7 days and [^3^H]thymidine incorporation was assed in the last 18 hours. Stimulation Index (S.I. = geomean CPM peptide/geomean CPM medium) of participants (n = 91) was calculated. (A) Prn- and Ptx-epitope responses with S.I.≥2 are indicated in blue for each individual participant. Participants and peptides were clustered on the number of positive responses, only participants responding to at least one of the peptides in our peptide panel are shown. The shades of blue indicate the magnitude (S.I. value) of the response (B) The combination of the clinical parameters age at blood donation and months after diagnosis in relation to responsiveness (Responsive = response with S.I. >2) to the tested (B) Prn- (n = 4) and (C) Ptx-peptides (n = 3) are depicted for (ex-)pertussis patients (n = 62). The degree of Prn- and Ptx- epitope responsiveness (0, 1, 2, 3 or 4 epitopes) per patient are indicated in shades of gray. The numbers in the quadrant insert indicate the percentage and ratio of epitope responsiveness (red) versus no epitope responsiveness (black) in the corresponding study populations. The odds ratio (OR) gives an indication whether differences in the number of responses between recently infected and not recently infected persons depends on age.

Besides adding to the insight in the presence, quality and maintenance of single epitope-specific CD4^+^ T cells in (ex-)pertussis patients in general, our peptide panel may provide information on the breadth of the Prn- and Ptx-specific response at the single donor level. An overview of the distribution and an indication of the lymphoproliferative capacity of the Prn- and Ptx-epitope specific responses in the (ex-)pertussis patients and household contacts from our clinical study (Figure 1) is shown in Figure 6A. Only participants responding to at least one of the peptides in our peptide panel and responses with S.I.≥2 are indicated and categorized according to S.I. value. Participants and peptides were clustered on the number of positive responses. Among responders to any of the peptides (58,2% of 91 donors), most of the participants responded to 1 or 2 peptides (28.3% (15/53) and 37.7% (20/53) respectively) from our panel (Figure 6A). However, there were also donors that responded to more than 1 or 2 of the 7 peptides in our panel, indicating that at the donor level pertussis-epitope multispecificity does exist (Figure 6A).

Since we included (ex-)pertussis patients at various time points after *B. pertussis* infection and at various ages, the confirmed pertussis patients could be divided in recently infected (<3 months after diagnosis) vs ex-pertussis (≥3 months after diagnosis) patients and based on their age (at time of blood sampling) (Figure 1). A patient was indicated an epitope-specific responder when S.I. of that response was ≥2. The odds ratio (OR) gives an indication whether differences in the number of responses between recently infected and not recently infected patients depends on age. In recently infected patients, the frequency of (multi-)Prn- or Ptx- epitope ‘responders’ is comparable in younger (<30 years of age) vs older patients (Figure 6B and C). Notably, in young ex-pertussis patients the extent of Ptx-epitope responsiveness is significantly higher than in older ex-pertussis patients (p = 0.0038) (Figure 6C). In fact, in young patients the percentage of (multi-)Ptx-epitope ‘responders’ seems unaffected with time after infection. For the Prn-epitope responses, a similar trend was detected (p = 0.073) (Figure 6B). In the older ex-pertussis patients no multi-epitope Prn- or Ptx-responsiveness was observed (Figure 6B and C). This indicates that waning immunity through loss of breadth of the pertussis response is more prominent in older ex-pertussis patients, while long-term persistence of multi-epitope responses for the Prn- and Ptx-peptide panel seems to occur in ex-pertussis patients of younger age.

## Discussion

Analysis of *B. pertussis*-specific (memory) CD4^+^ T cell responses at the epitope-level can shed light on the basis of waning immunity against this pathogen. Therefore we selected four Prn- and three Ptx- CD4^+^ T cell epitopes and compared direct *ex vivo* lymphoproliferative capacity, quality and breadth of epitope-specific CD4^+^ T cell responses, cross-sectionally in various groups of (ex-)pertussis patients and household contacts. The selected peptide panel appeared a useful tool to characterize Prn- and Ptx-specific CD4^+^ T cell responses at the single specificity level, since all selected Prn- and Ptx-peptides were immunogenic (Figure 2) and subtle differences in the breadth of the pertussis specific response were identified (Figure 6B and C). Since the peptides used are proven CD4^+^ T cell epitopes (Table 1) and most responses contained typical combinations of CD4^+^ T cell cytokines (Figure 4A), CD4^+^ T cells are most likely the responder cells involved. Unfortunately, we did not have access to additional fresh patients' material to perform CD4 blocking or depletion experiments to formally prove this assumption. In rare healthy donors with fresh ex vivo epitope responsiveness, we could block P.69 Prn_7–30_, PtxS1_141–158_ and PtxS1_189–206_ specific lymphoproliferative responses and cytokine production by anti-CD4 mAbs or CD4 T cell depletion, but not by anti-CD8 mAbs or CD8 T cell depletion (Figure S3 and S4), confirming our hypothesis. More donors responding to the other epitopes need to be identified to extend these blocking studies.

Together the epitope responses detected in half of the Prn- and Ptx-protein responsive participants revealed various characteristics. First, we found that the percentage Ptx-peptide responsiveness was higher than the Prn-peptide responsiveness (Fig. 2B and 6A). Furthermore, the Ptx-epitope specific responses showed higher proliferation capacity (S.I.) compared to Prn-epitope specific responses (Figure 2A). The observation could be due to the binding strength of the peptides to HLA-molecules. However, binding prediction of our peptide panel is only partially related with lymphoproliferative responsiveness. The Ptx-peptides, having the broadest coverage of HLA-DR alleles based on predicted bindingscores (Figure S1) indeed had the highest responsiveness in lymphoproliferation assays (Figure 2). Peptide Prn_583–606_, having the next broadest bindingscore in rank, did however yield the least responsiveness. Thus, predicted binding strength to HLA is not the only factor determining immunogenicity. Protein expression, inter-peptide competition and differential peptide processing also play a role in antigen-presentation and therefore also dictate immunogenicity [37]–[39]. Furthermore on the CD4^+^ T cell side, higher lymphoproliferative responsiveness can be due to higher precursor frequency or intrinsic proliferative capacity of peptide-specific T cells, partly determined by TCR affinity and the competition between T cells for interaction with APCs [40], [41]. These issues might be addressed with the help of MHC class II peptide tetramers, some of which are currently under development.

Secondly, in contrast to the magnitude of Ptx-epitope specific proliferative responses, Ptx protein responsiveness is declining over time after infection (Figure 3). This outcome is likely due to the fact that the Ptx-protein response is the sum of an unknown number of epitope responses. Apparently, Ptx epitopes that are not included in the panel, seem to contribute especially to early responses after diagnosis. The time-dependent declining lymphoproliferative responsiveness to whole antigen was specific for Ptx, since lymphoproliferation to Prn seems quite stable. Natural boosting by sub-clinical pertussis infection might play a role in the different maintenance of Prn- and Ptx-specific memory CD4^+^ T cells. Since P.69 Prn is expressed by multiple Bordetella species and other infectious agents, the exposure to P.69 Prn might be more frequent than to Ptx [42], [43]. Furthermore, P.69 Prn and Ptx have different expression patterns in the *B. pertussis* infection, since P.69 Prn is a surface-associated molecule involved in the adherence phase of the bacteria to respiratory epithelial cells, and Ptx is an extracellular toxin expressed and released only after colonization. These differences may affect the exposure-duration and -environment of immune cells to the different antigens during sub-clinical pertussis infection, perhaps resulting in Prn-specific lymphoproliferative responses which are quite stable over time after infection, while the lymphoproliferative capacity of Ptx-protein responses are not. However, this phenomenon needs to be further elucidated.

Thirdly, we demonstrate that pertussis epitope-specific T cell responses contained a Th1 and a Th2 component irrespective of the epitope studied or time after infection (Figure 4). We hardly found any CD4^+^ T cells simultaneously producing Th1 and Th2 cytokines (Figure 5). However the fact that within a single epitope-specificity distinct Th cytokine profiles are found, suggests some degree of flexibility in the pertussis-specific CD4^+^ T cell offspring. Recent genomic studies indicate that, although on a superficial level similar Th2 responses are observed, the gene expression network is different for pertussis-specific CD4^+^ T cells (induced by acellular pertussis vaccination) compared to e.g. allergen-specific CD4^+^ T cells. While the gene expression profile of atopy-specific CD4^+^ T cells are completely Th2, the gene expression profile of pertussis-specific CD4^+^ T cells is besides Th2 also Th1 [44]. Thus it seems that the pertussis response is not completely fixed and can still be steered into a more favorable Th1 profile, which is important knowledge in view of vaccination strategies. Future research in murine pre-clinical models should elucidate the potential to re-program pertussis-specific T cell responses.

Finally, analysis of individual (ex-)pertussis patients revealed that multi-epitope specificity long-term after infection was lower in patients ≥30 yrs of age, than in patients <30 yrs of age (Figure 6B and C). In general, vaccine type cannot account for these differences since both age groups received wP in their primary vaccination series according to the Dutch National Immunization Programme. However, patients older than 58 yrs of age, were born before wP vaccination was introduced. Therefore, it cannot be excluded that lack of priming by vaccination attributes to absence of multi-epitope specificity in three non-vaccinated older ex-pertussis patients. However, perhaps more likely, aging of the CD4^+^ T cell compartment could play a role in the lack of multi-epitope specificity in older ex-pertussis patients. Impairment of human CD4^+^ T cell responsiveness to stimulation was associated with higher age [45]. Sharma et al showed phenotypical distinctions between pertussis-specific CD4^+^ T cells in vaccinated infants and adults. Compared to the pertussis-specific memory CD4^+^ T cells in infants, relatively more cells in adults were shown to have an end-stage CCR7^−^CD27^−^ differentiation memory phenotype [46]. CCR7^−^CD27^−^ memory CD4^+^ T cells have been associated with shorter telomeres and decreased proliferative capacity, compared to early-stage CCR7^+^CD27^+^ differentiated memory CD4^+^ T cells [47]. Therefore, partial loss of anti-pertussis CD4^+^ T cell specificities with age, may reflect intrinsic immunosenescence. More research, including larger patients groups and single cell analysis tools, is needed to confirm and identify insufficiencies in the maintenance phase of the adaptive immune response to *B. pertussis* throughout life.

In summary, our study provides more insight in the quality and maintenance of pertussis-specific CD4^+^ T cells in (ex-)pertussis patients. These data show that CD4^+^ T cells responding at the single epitope level to a common pathogen such as *B. pertussis* may comprise a Th1 and Th2 component, suggesting that responding cells are not fixed to one lineage and are characterized by flexibility. The breadth of the pertussis-specific CD4^+^ T cell response seems dependent on age and time after infection. Loss of multi-epitope specificity in memory pertussis CD4^+^ T cell responses could play a role in waning effectiveness of pertussis immunity in older age groups. These observations can have implications for vaccination strategies and vaccine development. Steering towards an anti-bacterial Th1 profile and improving the breadth and long-term proliferative capacity of pertussis CD4^+^ T cell responses may elongate immunity against pertussis.

## Materials and Methods

### Ethics Statement

This study was conducted according to the principles expressed in the Declaration of Helsinki. The study was approved by the accredited Review Board STEG (Stichting Therapeutisch Evaluatie Geneesmiddelen) and is currently managed by the METC UMC Utrecht (Medisch Ethische Toetsingscommissie Universitair Medisch Centrum Utrecht) (CCMO nr: NL16334.040.07). The practicability of the study in the collaborating hospitals was accorded by their Review Boards. All participants provided written informed consent for the collection of samples and subsequent analysis. Written informed consent for minor participants was provided by both parents of the participants.

### Clinical cohort and isolation of PBMC

For this study blood samples were collected from ninety one volunteers as part of a cross-sectional observational study investigating pertussis specific immunity in the general Dutch population (NVI-243, NL16334.040.07). The participants were symptomatic pertussis patients recruited at a known time interval after their laboratory confirmed pertussis infection based on the diagnosis information provided by General Practitioners and Pediatricians, and household contacts of symptomatic patients within one month of diagnosis. Peripheral blood mononuclear cells (PBMC) were isolated from blood samples on the day of blood collection by centrifugation in Vacutainer cell preparation tubes (CPT, Becton and Dickinson) containing sodium citrate. PBMC were used directly, except in indicated cases after cryopreservation (in 80%FCS/20%DMSO). For analysis, participants were stratified according to study population (confirmed pertussis patients; household contacts), age (<30 yrs of age; ≥30 yrs of age) and/or time after diagnosis (t.a.d.<3 mnths; t.a.d. ≥3 mnths), respectively, as summarized in Figure 1. All patients included in the study received a primary serie of whole cell vaccine (wP) according to the Dutch National Immunization Programme, except voluntarily non-vaccinated patients (n = 2 in the group <30 yrs of age and t.a.d. ≥3 mnths) and patients born before 1953 (n = 7 in the group ≥30 yrs of age and t.a.d. <3 mnths, and n = 3 in the group <30 yrs of age and t.a.d. >3 mnths).

### Prn and Ptx antigens and peptides

Recombinant *B. pertussis* P.69 Prn1 (Prn) was expressed and purified from an *E. coli* construct as described in literature [48]. Purified *B. pertussis* Ptx was purchased commercially (Kaketsuken, Japan). Molecular weight and purity of these antigens were verified using SDS-PAGE, and the presence of detectable impurities of *E. coli* LPS in P.69 Prn or *B. pertussis* LOS in Ptx, respectively, was ruled out in a Limulus Amebocyte Lysate (LAL) test (hence endotoxin levels were <0.015 EU/ml). Synthetic Prn and Ptx peptides, encompassing CD4^+^ T cell epitopes from P.69 Prn1 and PtxS1 source proteins, respectively, were prepared by FMOC solid phase synthesis using a SYRO II simultaneous multiple peptide synthesizer (MultiSyntech GmbH, Witten, Germany). The purity and identity of the synthesized Prn and Ptx peptides was assessed by reverse phase high performance liquid chromatography (HPLC) and was >70% pure.

### In silico prediction of epitopes

ProPred, a MHC class II binding prediction server, locates within a protein sequence promiscuous binding regions by common HLA-DR alleles (http://www.allelefrequencies.net) [49]. The aminoacid sequences of P.69 Prn1 and PtxS1 were submitted to the server and the predicted binding scores of the selected Prn- and Ptx-peptides were identified.

### Proliferation assay

The presence of proliferating epitope-specific CD4^+^ T cells was assessed by [^3^H]thymidine incorporation. Freshly isolated PBMC (10^5^ cells per well in 96-well round-bottom plates) were stimulated with individual synthetic peptides representing Prn and Ptx epitopes at 1 µM (Table 1), whole Prn or Ptx protein at 1 µg/ml (3 or 6 wells per condition), PHA at 1 µg/ml as a positive control and medium (AIM-V (Gibco)/2% human AB serum (Sanbio/Harlan)) as negative control for 6 days at 37°C. At day 6, 100 µl supernatant volumes per well were removed for cytokine analysis. Then 0.5 µCi [^3^H]thymidine was added to the culture 18 hours before harvesting the cells and [^3^H]thymidine incorporation was determined as counts per minute (CPM) with LKB/Wallac 1205 Betaplate Liquid Scintillation Counter. Every sample investigated showed proliferation to PHA, indicating good viability and polyclonal T cell responsiveness. The stimulation index (S.I.) was calculated for all responses as [geomean CPM of 3 or 6 wells in the presence of peptide or protein/geomean CPM of 3 control wells in the presence of medium only]. For the categorization as a positive epitope-specific response an S.I.≥2 was considered as a cut-off.

### Cytokine profiling

Concentrations of cytokines in culture supernatants representative for epitope-specific lymphoproliferative responses were determined using Bio-plex human Th1/Th2 and Th17 cytokine luminex kits (Bio-rad), according to manufacturer's instructions. For this, individual wells representing a donor's PBMC culture in the presence of a single peptide were first scored for lymphoproliferation, and supernatants from wells with S.I.≥2 were analyzed. Supernatants were taken at day 6 after in vitro restimulation. Day 6 supernatant is optimal for analyzing Th cytokines, except for the early cytokine IL-2 and IL-4 (own obervations). Data acquisition was performed on a Biorad Bio-Plex200. Background cytokine levels released in the presence of medium only were subtracted from cytokine levels released after peptide stimulation.

### Fluorospot

Using frozen PBMC, cytokine production of Prn- and Ptx- epitope-specific CD4^+^ T cells was also determined in a dual IFNγ/IL-13 Fluorospot (Mabtech). The five participants analyzed in Figure 5, were selected based on positive epitope response and availability of PBMC. Frozen PBMC (2×10^5^ cells per well in 96-well fluorospot plates) were thawed and stimulated (in quadruplicate) with 1 µM individual Prn- or Ptx-peptides or medium (AIM-V (Gibco)/2% human AB serum (Sanbio/Harlan), in the presence of anti-CD28 monoclonal (1 µg/ml) for 48 hours at 37°C. At 48 h, plates were developed according to the manufacturer's protocols. Plates were analyzed on an AID iSpot reader using AID ELISpot software (AID diagnostika). Specific cytokine producing cells were determined by calculating [spots peptide stimulated wells – average spots medium control wells]. Fluorospot is suitable for detection of rare cells [36].

### Statistical analysis

For data visualization GraphPad Prism (GraphPad Software) and R software (http://www.R-project.org/) was used. Differences in S.I. were analyzed with the nonparametric Mann-Whitney U test (Figure 2A). The association between the S.I. of proliferative responses and closeness to infection was assessed by linear regression, where the log(S.I.) was taken as the response variable and log(closeness to infection) was taken as explanatory variable, respectively. The slope of the regression line was tested to be equal to zero using a t-test (Figure 3). The association between the cytokine responses and closeness to infection was assessed by linear regression, where the log(Th1) or log(Th2) was taken as the response variable and log(closeness to infection) was taken as explanatory variable, respectively. The trend was tested to be equal to zero using a t-test (Figure 4C). The odds ratio was calculated using Bayesian logistic regression [50], where the number of responders was taken as the response variable and time after infection (<3 months and ≥3 months), age (<30 years and ≥30 years) and their interaction as explanatory variables. The odds ratio of the interaction term is shown in Figure 6B and C.

## Supporting Information

Figure S1 **P.69 Prn_7–24_ and PtxS1_141–158_ activate specific human CD4^+^ T cell clones in a HLA-DR restricted manner.** P.69 Prn- (A) and PtxS1-peptide (B) specific proliferation was determined by [^3^H]thymidine incorporation after 2 days of stimulation with various concentration of peptides (Left panels) in the presence or absence of α-HLA-DR or α-HLA-DQ monoclonal antibodies (Right panels). [^3^H]thymidine incorporation was determined as counts per minute (CPM) with LKB/Wallac 1205 Betaplate Liquid Scintillation Counter. The stimulation index (S.I.) was calculated as [mean CPM in the presence of peptide/mean CPM in the presence of medium only].
(TIF)

Figure S2 **ProPred HLA-DR binding prediction for Prn- and Ptx-peptides.** The amino acid sequence of P.69 Prn and PtxS1 were submitted to the ProPred MHC class-II binding peptide prediction server. The peptides of the Prn and Ptx panel are shown in the order of their immunogenicity in Figure 6A, and the amino acid sequence with predicted binding to HLA-DR are displayed in the figure. For each HLA-DR allele a score is calculated for the predicted binding of that sequence, the colors indicate the strength of the predicted binding (as a percentage of the highest score that can be achieved by that HLA–DR allele).
(TIF)

Figure S3 **P.69 Prn_169–192_-specific proliferation and cytokine production in PBMC is CD4-dependent.** Freshly isolated PBMC (10^5^ cells per well in 96-well round-bottom plates) were stimulated with P.69 Prn_169–192_-peptide at 1 µM (6 wells per condition) in the presence or absence of α-CD4 or α-CD8 monoclonal antibodies (both 1∶300 ascites with an average antibody concentration of 3–5 mg/ml), and medium (AIM-V (Gibco)/2% human AB serum (Sanbio/Harlan)) for 6 days at 37°C. At day 6, 100 µl supernatant volumes per well were removed and pooled for cytokine analysis. (A) [^3^H]thymidine incorporation was determined as counts per minute (CPM) with LKB/Wallac 1205 Betaplate Liquid Scintillation Counter. Epitope-specific lymphoproliferative responses are shown in two donors (B) Concentrations of cytokines in culture supernatants were determined using Bio-plex human Th1/Th2 and Th17 cytokine luminex kits (Bio-rad), according to manufacturer's instructions. The epitope-specific cytokine responses are shown in two donors. *p<0.05.
(TIF)

Figure S4 **Primarily CD4^+^ T cells produce cytokines in response to PtxS1-peptides and protein.** Freshly isolated PBMC were depleted for CD4^+^ or CD8^+^ cells by magnetic cell separation (MACS, Miltenyi Biotec) and resulting cell populations were viable and >95% pure as determined by Flowcytometry. Cells were stimulated (10^5^ cells per well in 96-well round-bottom plates) with PtxS1-peptides at 1 µM or Ptx protein at 1 µg/ml (6 wells per condition), and medium (AIM-V (Gibco)/2% human AB serum (Sanbio/Harlan)) for 6 days at 37°C. At day 6, 100 µl supernatant volumes per well were removed and pooled for cytokine analysis. Concentrations of cytokines in culture supernatants were determined using Bio-plex human Th1/Th2 and Th17 cytokine luminex kits (Bio-rad), according to manufacturer's instructions. The epitope-specific cytokine responses are shown in two donors.
(TIF)
