# Supplementary figures and images for: Loss of Multi-Epitope Specificity in Memory CD4+ T Cell Responses to B. Pertussis with Age
Source: PLoS One. 2013 Dec 31;8(12):e83583. doi: 10.1371/journal.pone.0083583 (PMC3877060; doi:10.1371/journal.pone.0083583)

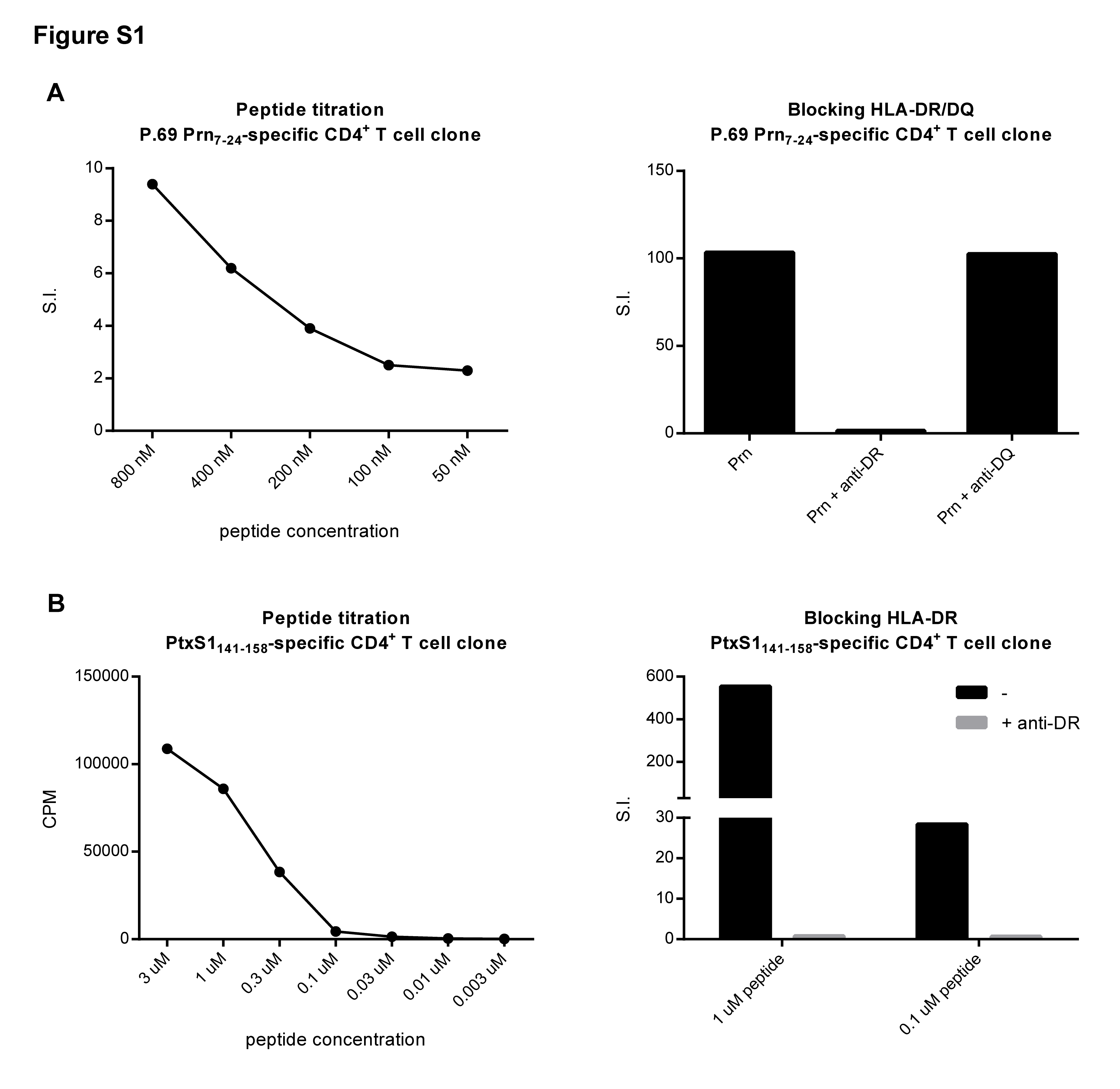

Supplement: Figure S1 — P.69 Prn7–24 and PtxS1141–158 activate specific human CD4+ T cell clones in a HLA-DR restricted manner. P.69 Prn- (A) and PtxS1-peptide (B) specific proliferation was determined by [3H]thymidine incorporation after 2 days of stimulation with various concentration of peptides (Left panels) in the presence or absence of α-HLA-DR or α-HLA-DQ monoclonal antibodies (Right panels). [3H]thymidine incorporation was determined as counts per minute (CPM) with LKB/Wallac 1205 Betaplate Liquid Scintillation Counter. The stimulation index (S.I.) was calculated as [mean CPM in the presence of peptide/mean CPM in the presence of medium only]. (TIF) [file pone.0083583.s001.tif]

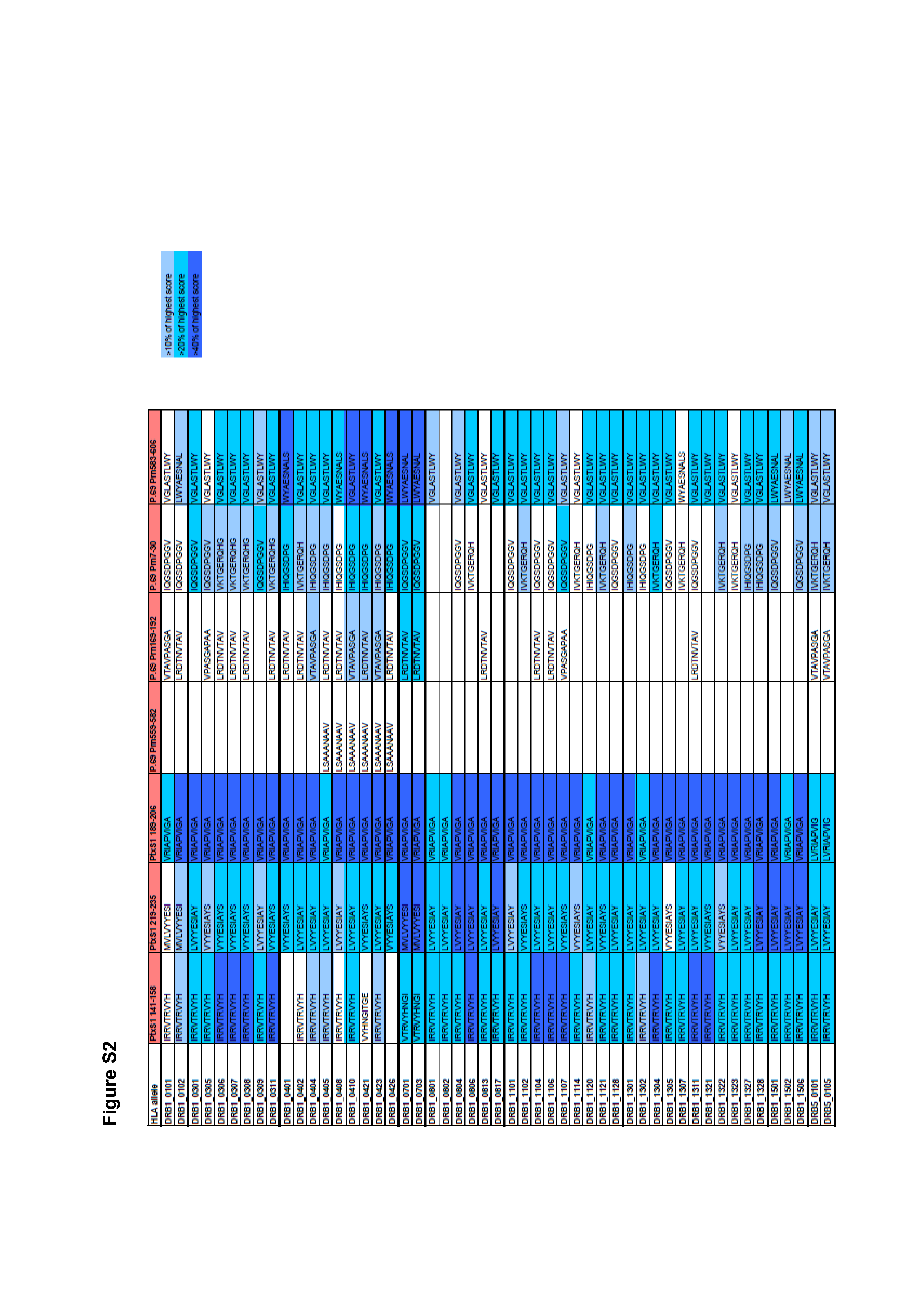

Supplement: Figure S2 — ProPred HLA-DR binding prediction for Prn- and Ptx-peptides. The amino acid sequence of P.69 Prn and PtxS1 were submitted to the ProPred MHC class-II binding peptide prediction server. The peptides of the Prn and Ptx panel are shown in the order of their immunogenicity in Figure 6A, and the amino acid sequence with predicted binding to HLA-DR are displayed in the figure. For each HLA-DR allele a score is calculated for the predicted binding of that sequence, the colors indicate the strength of the predicted binding (as a percentage of the highest score that can be achieved by that HLA–DR allele). (TIF) [file pone.0083583.s002.tif]

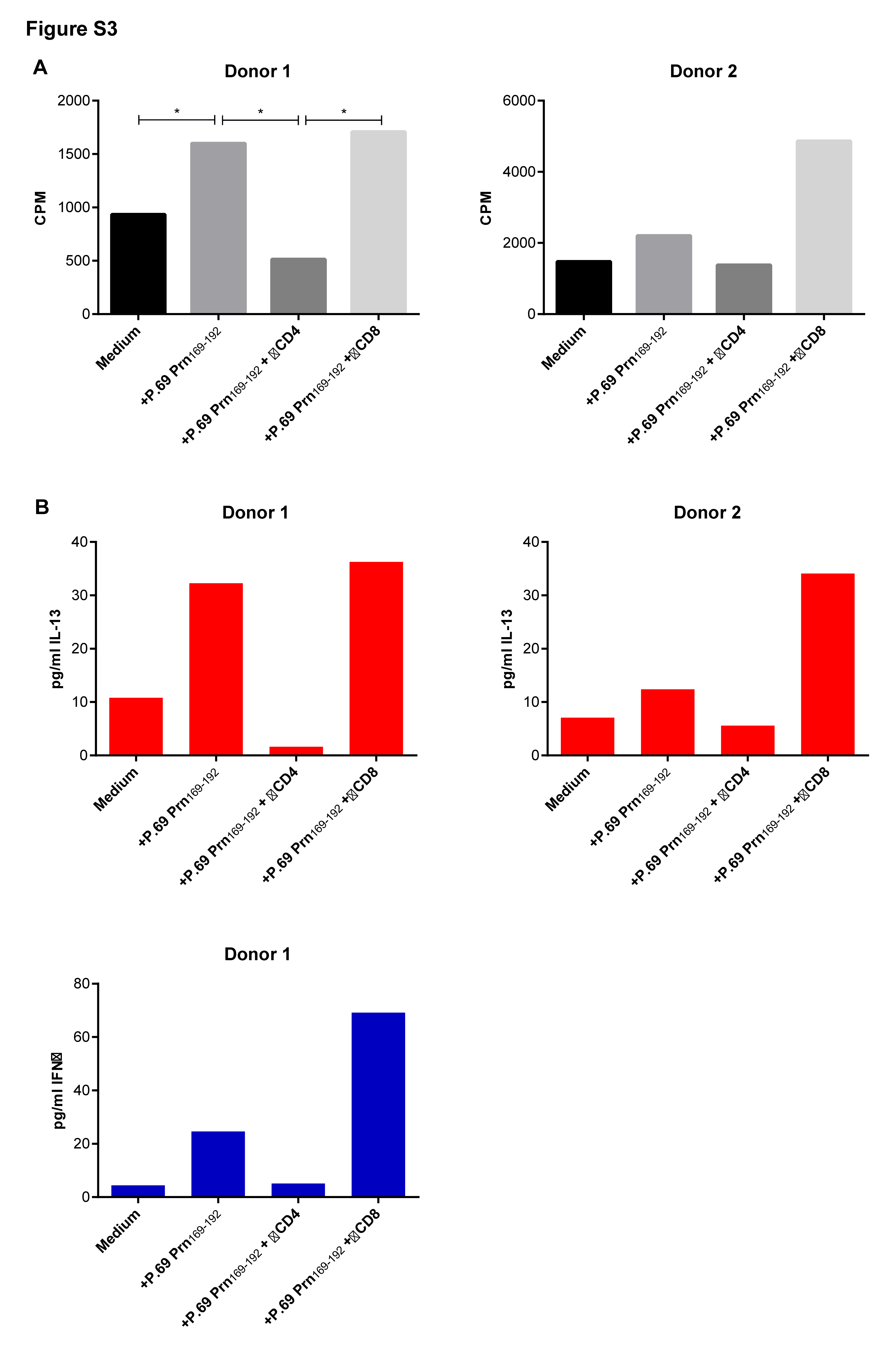

Supplement: Figure S3 — P.69 Prn169–192-specific proliferation and cytokine production in PBMC is CD4-dependent. Freshly isolated PBMC (105 cells per well in 96-well round-bottom plates) were stimulated with P.69 Prn169–192-peptide at 1 µM (6 wells per condition) in the presence or absence of α-CD4 or α-CD8 monoclonal antibodies (both 1∶300 ascites with an average antibody concentration of 3–5 mg/ml), and medium (AIM-V (Gibco)/2% human AB serum (Sanbio/Harlan)) for 6 days at 37°C. At day 6, 100 µl supernatant volumes per well were removed and pooled for cytokine analysis. (A) [3H]thymidine incorporation was determined as counts per minute (CPM) with LKB/Wallac 1205 Betaplate Liquid Scintillation Counter. Epitope-specific lymphoproliferative responses are shown in two donors (B) Concentrations of cytokines in culture supernatants were determined using Bio-plex human Th1/Th2 and Th17 cytokine luminex kits (Bio-rad), according to manufacturer's instructions. The epitope-specific cytokine responses are shown in two donors. *p<0.05. (TIF) [file pone.0083583.s003.tif]

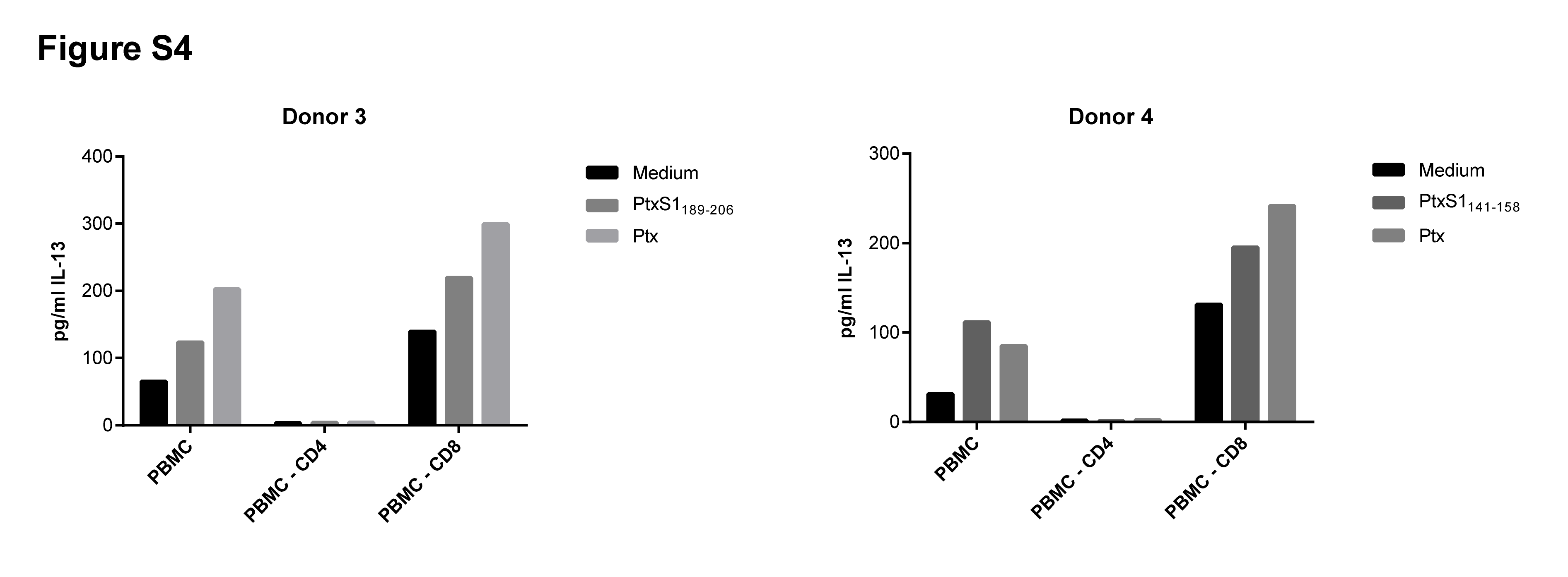

Supplement: Figure S4 — Primarily CD4+ T cells produce cytokines in response to PtxS1-peptides and protein. Freshly isolated PBMC were depleted for CD4+ or CD8+ cells by magnetic cell separation (MACS, Miltenyi Biotec) and resulting cell populations were viable and >95% pure as determined by Flowcytometry. Cells were stimulated (105 cells per well in 96-well round-bottom plates) with PtxS1-peptides at 1 µM or Ptx protein at 1 µg/ml (6 wells per condition), and medium (AIM-V (Gibco)/2% human AB serum (Sanbio/Harlan)) for 6 days at 37°C. At day 6, 100 µl supernatant volumes per well were removed and pooled for cytokine analysis. Concentrations of cytokines in culture supernatants were determined using Bio-plex human Th1/Th2 and Th17 cytokine luminex kits (Bio-rad), according to manufacturer's instructions. The epitope-specific cytokine responses are shown in two donors. (TIF) [file pone.0083583.s004.tif]
